# EHMTI-0077. Obesity-related intracranial hypertension in the rat – a possible idiopathic intracranial hypertension (IIH) model?

**DOI:** 10.1186/1129-2377-15-S1-F29

**Published:** 2014-09-18

**Authors:** M Uldall, D Bhatt, C Kruuse, M Juhler, I Jansen-Olesen, R Jensen

**Affiliations:** 1 Research Institute Glostrup, Danish Headache Center, Glostrup, Denmark; 2 Department of Neurology, Herlev Hospital, Herlev, Denmark; 3 Department of Neurosurgery, The National Hospital Rigshospitalet, Copenhagen, Denmark; 4 Department of Neurology, Danish Headache Center, Glostrup, Denmark

## Background

Idiopathic intracranial hypertension (IIH) is a condition of increased intracranial pressure (ICP) without identifiable cause. The majority of IIH patients are obese, which suggests a connection between CSF regulation and obesity. However, the pathophysiological mechanisms remain widely unresolved.

## Aim

To develop a long-term ICP monitoring method and investigate ICP in lean and obese rats. We also aimed to clarify if any ICP difference could be attributed to changes in some well-known ICP modulators; retinol and arterial partial pressure of CO2 (pCO2).

## Methods

ICP was measured in six obese and six lean Zucker rats with a newly developed epidural ICP monitoring method over a period of 31 days. Furthermore, arterial pCO2 and serum retinol were measured in blood samples from each animal.

## Results

Obese rats had significantly elevated ICP-levels compared to lean controls on all recording days (p < 0.0001). Serum retinol (lean: 10.54 ± 0.36, obese: 11.70 ± 0.91, p = 0.35) and arterial pCO2 (lean: 37.17 ± 1.58, obese: 41.25 ± 1.80, p = 0.16) did not differ between the two groups.

## Conclusion

Obesity-related intracranial hypertension in rats is not related to altered pCO2 levels or retinol metabolism. This indicates that the increase in ICP might be related to molecular changes in the brain caused by the adipose state. Although further studies are warranted, obese Zucker rats could potentially constitute a model for IIH.

No conflict of interest.

